# A Chromosome-Level Genome Assembly of Chiton *Acanthochiton rubrolineatus* (Chitonida, Polyplacophora, Mollusca)

**DOI:** 10.3390/ani14213161

**Published:** 2024-11-04

**Authors:** Jiangyong Qu, Xiaofei Lu, Chenen Tu, Fuyang He, Sutao Li, Dongyue Gu, Shuang Wang, Zhikai Xing, Li Zheng, Xumin Wang, Lijun Wang

**Affiliations:** 1 College of Life Science, Yantai University, Yantai 264005, China; qjy@ytu.edu.cn (J.Q.); luxiaofei18@outlook.com (X.L.); 18055456097@163.com (C.T.); 19554539611@163.com (F.H.); z123xcvbnm0709@163.com (S.L.); 19554539562@163.com (D.G.); wangshuang0456@126.com (S.W.); xingzhk@ytu.edu.cn (Z.X.); 2 First Institute of Oceanography, Ministry of Natural Resources, Qingdao 266061, China; zhengli@fio.org.cn

**Keywords:** *Acanthochiton rubrolineatus*, Polyplacophora, genome assembly, phylogeny, chromosome

## Abstract

(1) Background: Chitons (Mollusca, Polyplacophora) are relatively primitive species in Mollusca that allow the study of biomineralization. Although mitochondrial genomes have been isolated from Polyplacophora, there is no genomic information at the chromosomal level; (2) Methods: Here we report a chromosome-level genome assembly for *Acanthochiton rubrolineatus* using PacBio (Pacific Biosciences, United States) reads and high-throughput chromosome conformation capture (Hi-C) data; (3) Results: The assembly spans 1.08 Gb with a contig N50 of 3.63 Mb and 99.97% of the genome assigned to eight chromosomes. Among the 32,291 predicted genes, 76.32% had functional predictions. The divergence time of Brachiopoda and Mollusca was ~550.8 Mya (million years ago), and that of *A. rubrolineatus* and other mollusks was ~548.5 Mya; (4) Conclusions: This study not only offers high-quality reference sequences for the Acanthochiton rubrolineatus genome, but also establishes groundwork for investigating the mechanisms of Polyplacophora biomineralization and its evolutionary history. This research will aid in uncovering the genetic foundations of molluscan adaptations across diverse environments.

## Simple Summary

This research achieved the first chromosome-level genome assembly of *Acanthochiton rubrolineatus* (*A. rubrolineatus*), revealing a genome size of 1.08 Gb via PacBio long-read sequencing and high-throughput chromosome conformation capture (Hi-C). This milestone is crucial for exploring mollusk biomineralization. Out of 32,291 identified genes, 76.32% received functional annotations. The study revealed 248 genes that displayed significant positive selection (*p* < 0.05) in this species. These genes are notably involved in immune response, metabolic pathways, signaling, and biomineralization. Their functional changes suggest that *A. rubrolineatus* may have optimized its survival adaptations through evolutionary processes. Additionally, the investigation estimated that *A. rubrolineatus* diverged from other mollusks around 548.5 million years ago. This discovery is vital for comprehending mollusk-specific adaptive evolution.

## 1. Introduction

As an important branch of Mollusca, all species in the Polyplacophora, or chitons, were marine organisms. This group includes approximately 940 living species [1] and 430 [2] fossil species. From the Cretaceous to the present time, morphological changes in chitons have been minimal, serving as typical examples of “living fossils”. More than 10 species of chitons inhabit the coastal areas of China, with *A. rubrolineatus* [3] (Chitonida; Acanthochitonina; Acanthochitonidae; Acanthochitona) being the most common. *A. rubrolineatus* ranges from the Bohai Sea to the South China Sea. It dwells in the intertidal zone and on exposed rocks of low-tide areas.

Mollusks have long served as models to study the genetic mechanisms linked to biomineralization because they produce a wide variety of materials for shells, spines, scales, and teeth. Chitons biomineralize their teeth using a unique blend of materials. Most mollusks possess a feeding organ called the radula, equipped with rows of teeth composed of chitin. Many species harden these teeth with minerals like calcium carbonate or silica. In contrast, chitons harden their teeth with calcium phosphate (specifically, apatite) and cap each tooth with magnetite to enhance its cutting edge [4]. These iron coatings enable chitons to scrape algae from rocks without rapidly dulling or damaging their teeth. Moreover, chitons generate new teeth throughout their lives, forming a new row every three days [5,6]. To produce new teeth, chitons continuously sequester and transport significant quantities of iron [5,7,8], a considerable challenge since free iron induces oxidative stress [9]. Isolating the entire genome sequence of *A. rubrolineatus* will facilitate further investigation into the mineralization mechanism of the radula and enhance our understanding of the genetic mechanisms of biomineralization.

Magnetite, or nano-Fe_3_O_4_, has many potential applications, particularly due to its remarkable magnetic and surface effects. Among various applications, nano-Fe_3_O_4_ may serve in targeting drug carriers, immunoassays, and magnetic sensors. The presence of magnetic minerals in organisms was a widespread phenomenon. Since the last century, researchers have discovered magnetite in bacteria [10], insects [11], mollusks [12], fish [13], birds [14], and humans. Among these organisms, chitons, noted for their ability to biomineralize teeth, have remained a consistent focus of investigation [15]. Specifically, how to leverage the biomineralization mechanism to synthesize nanomaterials using simple methods has become a prominent topic for researchers.

In the absence of definitive fossil records, genomic reconstruction through comparisons of existing mollusk genomes is essential for understanding early mollusk ancestors and their evolution [16,17]. However, comprehending the genome information of mollusk ancestors proves challenging, as early genome sequencing is primarily centered on Bivalvia and Gastropoda. While chitons hold a vital position studying mollusk origin [18], most current knowledge on Polyplacophora exists at the mitochondrial level. Fourteen complete mitochondrial genomes of Polyplacophora have been documented, including *Katharina tunicate* [19], *Nierstraszella lineata* [20], *Acanthochitona avicula* [20], *Chiton albolineatus* [20], *Dendrochiton gothicus* [20], *Hanleyella oldroydi* [20], *Leptochiton nexus* [20], *Chaetopleura apiculate* [21], *Nuttallina californica* [21], *Cryptochiton stelleri* [21], *Cyanoplax caverna* [21], *Sypharochiton pelliserpentis* [22], *Sypharochiton sinclairi* [22], and *Lepidozona coreanica* (GenBank: MT070411.1). It has been statistically found that only 11 species have been assembled at the chromosome level: *Achatina immaculata*, *Archivesica marissinica*, *Biomphalaria glabrata*, *Crassostrea gigas*, *Crassostrea virginica*, *Chrysomallon squamiferum*, *Octopus sinensis*, *Pecten maximus*, *Pinctada mbricata*, *Pomacea canaliculata*, and *Sinonovacula constricta* (Table S1). In contrast to sequencing assembly strategies for other mollusks, the genome of *A. rubrolineatus* was constructed at the chromosome level from DNA samples extracted from the same individual and integrating short sequence, PacBio long sequence, and Hi-C sequencing data. This chromosome-level genome will not only enhance research on mollusk development and evolution, but also offer critical resources for understanding biomineralization in *A. rubrolineatus* and other mollusks.

## 2. Materials and Methods

### 2.1. Sample Collection, Library Construction, and Whole-Genome Sequencing

**Figure 1:**
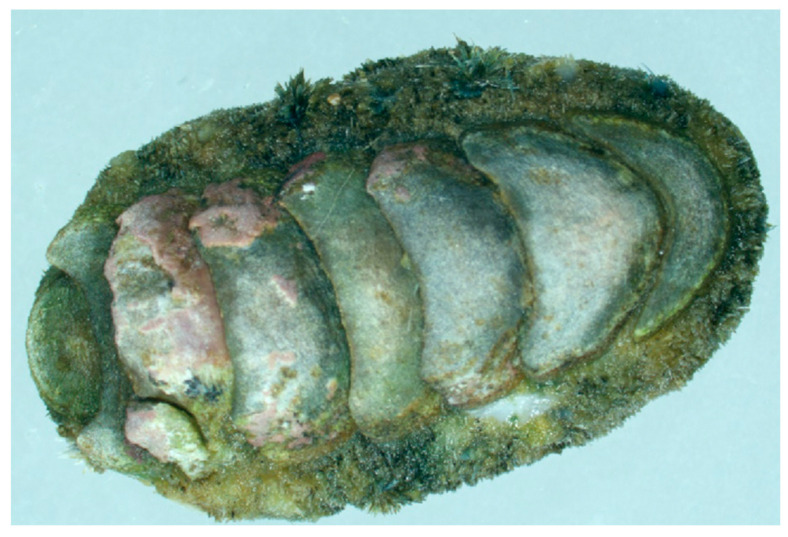
Picture of adult *A. rubrolineatus* sample.

The study utilized a specimen of *A. rubrolineatus* obtained from Zhifu Bay near Jingu Village, Yantai City, Shandong Province (Figure 1), from which high-molecular-weight DNA was extracted from the entire body using QIAamp^®^ DNA Mini Kit (OMEGA, Germany) according to the manufacturer’s protocol. Notably, prior to DNA extraction, we removed shell plates and tentacles (including small tentacles and nodules) from the entire body, retaining only the internal muscle tissue for DNA extraction. A PacBio Sequel II-CLR (continuous long reads) sequencing library was sequenced from muscle tissue cells with the PacBio Sequel system using version 3.0 chemistry. This resulted in 5,779,469 subreads, with a subread N50 of 33,471 kb and a total of 111,571,420,069 bp.

A Hi-C library was generated as described [23]. Briefly, high molecular weight DNA is assembled into chromatin in vitro and then chemically crosslinked with 1% formaldehyde prior to restriction digestion with a fixation time of approximately 10 min. The crosslinked DNA is digested using HindIII to produce DNA fragments with sticky ends; the ends are repaired by flattening, and the formaldehyde crosslinking is terminated using glycine to ensure that the crosslinking does not proceed excessively. Neighboring DNA fragments were ligated using DNA ligase; the DNA was digested using proteinase K to release the cross-links, purified and broken into fragments of 500–700 bp in length, and the labeled DNA was captured using affinity beads. The libraries were subsequently sequenced on the BGISEQ-500 platform, resulting in a total of 976.99 Mb of data (Table 1).

### 2.2. Genome Assembly the A. rubrolineatus

PacBio reads (10^3^× sequence coverage) were assembled using Canu (v. 2.1.1) [24]. Canu initiates error correction on raw long-read data, fixing sequencing errors. It discards low-quality reads and retains high-quality ones for assembly. An overlap map identifies read overlaps by comparing sequence similarities. The optimal path from this map constructs contiguous fragments (contigs). Duplicate regions are addressed by analyzing branches in the overlap graph. Spliced read segments form contiguous fragments, with ongoing error correction during splicing. The resulting assembly was polished using Pilon (v. 1.22), which used MiniMap2 to compare short reads to the assembly file. This process aimed to analyze the results and detect possible errors, such as SNP detection and correction, InDel correction, and gap filling [25]. Eventually, this produced 645 contigs with an N50 of 3,626,433 bp and a total genome size of 1.08 Gb (Table 1). The integrity of conserved gene regions in the *A. rubrolineatus* genome was predicted using BUSCO data from molluscs metazoa_odb9 (https://busco-data.ezlab.org/v5/data/lineages) (accessed on 2 October 2024). The broken assembly was then scaffolded with Hi-C data using the 3D-DNA (3D de novo assembly) pipeline (https://github.com/aidenlab/3d-dna) (accessed on 2 October 2024) [26].

### 2.3. Genomic Annotation

For annotation repetitive sequences, we employed RepeatModeler v1.0.10 (RepeatModeler, RRID:SCR 015027). This tool uses two complementary computational methods, RECON v1.08 and RepeatScout v1.0.5 (RepeatScout, RRID:SCR 014653) [27], to identify repeat element boundaries and family relationships from sequence data. Subsequently, the outputs from RepeatModeler and the RepBase library [28] were combined and used for further characterization of transposable elements (TEs), many of which are not repetitive, and other repeats by homology-based methods, including identification with Repeat-Masker (v4.0.7, rmblast-2.2.28) (RepeatMasker, RRID:SCR 012954). For structural annotation of genes, we predicted annotations de novo with AUGUSTUS (v3.2.3) [29], GlimmerHMM (v 3.0.4) [30] (Glimmer Hidden Markov Model), and Genscan [31]. We also employed Tritnity (v2.11.0) [32] for a de novo assembly of the transcriptome, followed by HISAT2 (v 2.2.1)-Stringtie (v 2.2.5) [33] (Hierarchical Indexing for Spliced Alignment of Transcripts2-Stringtie) and PASA (v 2.4.1)-Transdecoder (v 5.7.1) [34] (Program to Assemble Spliced Alignments Transdecoder) to predict transcripts. Additionally, we utilized Genewise (v2.4.1) [35] for homologous annotation with protein sequences downloaded from the NCBI (National Center for Biotechnology Information) (https://www.ncbi.nlm.nih.gov/) (accessed on 2 October 2024) database for the following seven molluscan species: *Aplysia californica*, *Argopecten purpuratus*, *Crassostrea gigas*, *Crassostrea virginica*, *Lottia gigantea*, *Pila canaliculata*, and *Scapharca broughtonii*. Finally, GLEAN software (v 1.0.1) was used to integrate the results obtained from the above three methods to obtain the final results.

For annotation of gene functions, we searched the predicted protein sequences identified in *A. rubrolineatus* within the TrEMBL [36], SwissProt (http://www.ebi.ac.uk/uniprot) (accessed on 2 October 2024) [37], KEGG (http://www.genome.jp/kegg/) (accessed on 2 October 2024) [38], GO (http://geneontology.org/) (accessed on 2 October 2024) [39], and InterProScan (https://www.ebi.ac.uk/interpro/) (accessed on 2 October 2024) [40] databases.

### 2.4. Genome Evolution

To investigate the evolutionary relationship of *A. rubrolineatus* and other species, we identified 34,597 gene families in total. Among these, 1808 gene families were unique to other species, identified through BLAST (Basic Local Alignment Search Tool) searches against the genomes of 15 species (*A. californica*, *B. glabrata*, *C. gigas*, *C. virginica*, *E. chlorotica*, *L. gigantea*, *M. yessoensis*, *N. schrenckii*, *O. bimaculoides*, *O. vulgaris*, *P. maximus*, *P. canaliculata*, *S. broughtonii*, *L. anatine*, *A. rubrolineatus*) with an e-value cutoff of 1 × 10^5^. The gene set data used were downloaded from Ensembl or NCBI. The genes obtained from databases with a frame shift or that encoded proteins of <50 amino acids were removed; for protein-coding genes with alternative splicing isoforms, only the longest protein sequence prediction was used for subsequent analysis. In this study, *A. rubrolineatus*, *A. californica*, *B. glabrata*, and *C. gigas* were selected for covariance analysis, which began with the use of Lastz (v1.04.15) to test for gene sequence homology between species, which has a longer comparison length while allowing for a small number of gaps in the genome. Then, we filtered out gaps > 1% of the aligned fragments and visualized the results using Circos software (v 0.69-9) [41].

We then identified 531 single-copy ortholog alignments and performed evolutionary analyses. Amino acid and nucleotide sequences of orthologous genes were aligned with the multiple alignment software MUSCLE (v 3.8.31) [42] with the default parameters. A maximum likelihood method tree was inferred based on the matrix of nucleotide sequences using RAxML (v8.2.12) [43] (Randomized Axelerated Maximum Likelihood), with default nucleotide substitutions as per the JTT model [44]. Clade support was assessed using the bootstrapping algorithm in the RAxML package with 100 alignment replicates. The species divergence time was inferred with MCMCTree (Markov Chain Monte Carlo Tree) included in PAML (v4.7a0) [45] with the parameter setting as ‘--model 0 --rootage 500 -clock 3’. According to the MCMCTree tutorials, we estimated the divergence times using the approximate method with fossil calibrations from Timetree (http://www.timetree.org) (accessed on 2 October 2024). Evolutionary analyses were performed using single-copy protein-coding genes from related mollusk species.

## 3. Results

### 3.1. Genome Sequencing and Assembly

The Polyplacophora genome differs in content and organization from conchiferan mollusk genomes. Using K-mer-based analysis, the estimated genome size of *A. rubrolineatus* is 1Gb. The *A. rubrolineatus* genome exhibited a heterozygosity rate of 1.14% (Table 2) and a repeat rate of 59.9%. High heterozygosity is often attributed to high rates of gene flow associated with broadcast spawning and far-dispersing larvae [46], and it is frequently noted as an obstacle to genome assembly in mollusks [47,48,49,50]. We obtained whole-genome sequencing data based on the PacBio sequencing platform with a total of 111.57 Gb. After genome assembly, de-redundancy, and error correction, the obtained genome size was 1.08 Gb, and the contig N50 was 3.63 Mb, which is 0.08 Gb larger than the estimated genome (Table 2). The genome of *A. rubrolineatus* is better than *Biomphalaria glabrata*, a gastropod mollusk. The genome size of *B. glabrata* is 916.388 Mb and the BUSCO (v4.1.2) (Benchmarking Universal Single-Copy Orthologs) is 89.10%.

**Figure 2:**
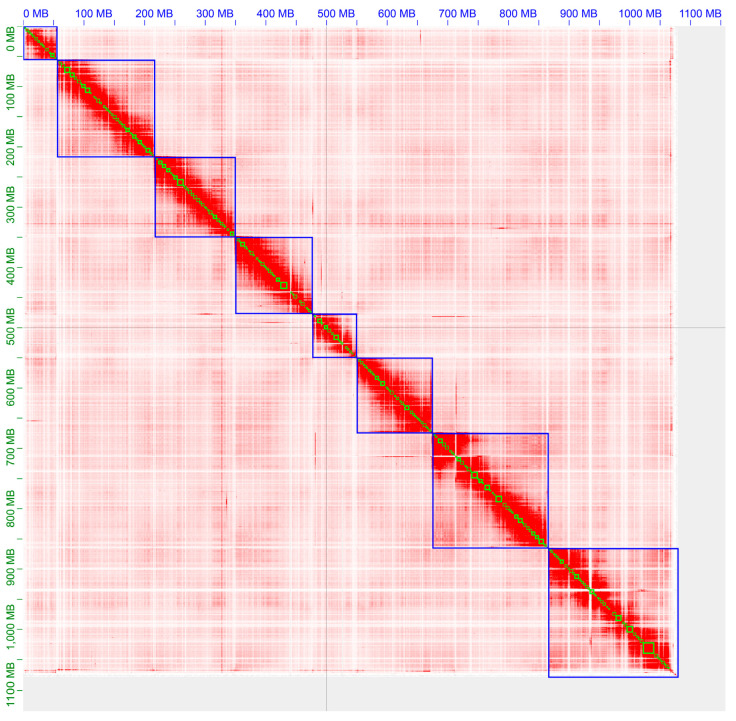
Thermogram of chromosome interactions in *Acanthochiton rubrolineatus* based on HI-C sequencing data. Blue boxes identify chromosomal regions. Both horizontal and vertical coordinates indicate the physical location on the chromosome, and the color gradient indicates the strength of the interaction.

**Figure 3:**
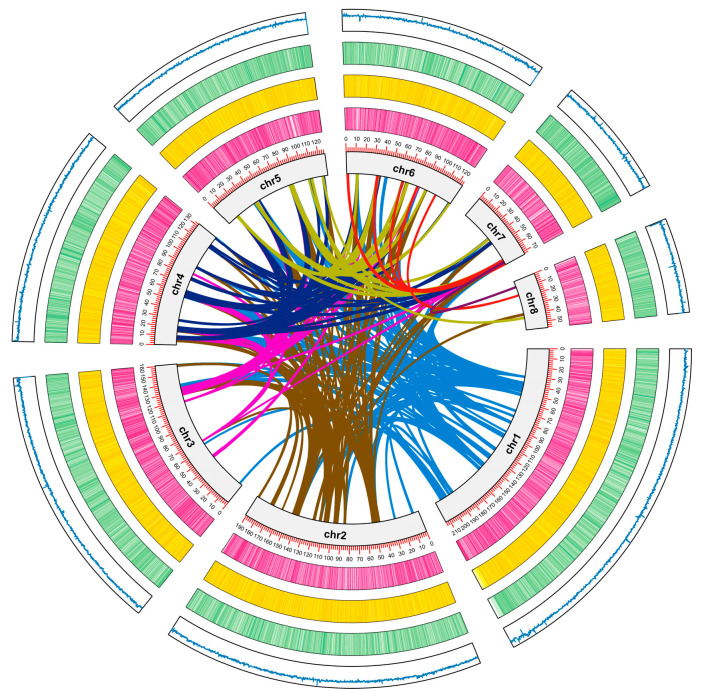
Genome Circos plot of *A. rubrolineatus.* Inner circle outward: genome covariance, chromosomes, gene distribution, TE(DNA) distribution, TE(LINE) distribution, GC content curves, Genome-wide covariance analysis of *A. rubrolineatus*, with *A. californica*, *B. glabrata*, and *C. gigas*. The lines connecting the different chromosome segments, which vary in color, indicate the interactions between the different chromosomes.

Genome size differences are the result of random genetic or gene combination processes that organisms can tolerate, such as polyploidization, spontaneous deletion or insertion (especially of introns), an increase in tandem repeat length, and an increase in the copy number of transposable elements (TEs). To further improve the continuity of the assembled genome and anchor the assemblies into chromosomes, we used Hi-C data to order and orient the contigs as well as to correct misjoined sections and merge overlaps. From this, we assembled eight chromosomes (Figure 2) of lengths ranging from 56.87 Mb to 214.36 Mb (Table 3 and Figure 3). The total length of the sequences mounted on the chromosomes accounted for 99.97% of the total genome length. This study thus provides the first chromosome-anchored genome of Polyplacophora.

### 3.2. Genome Quality Evaluation

**Figure 4:**
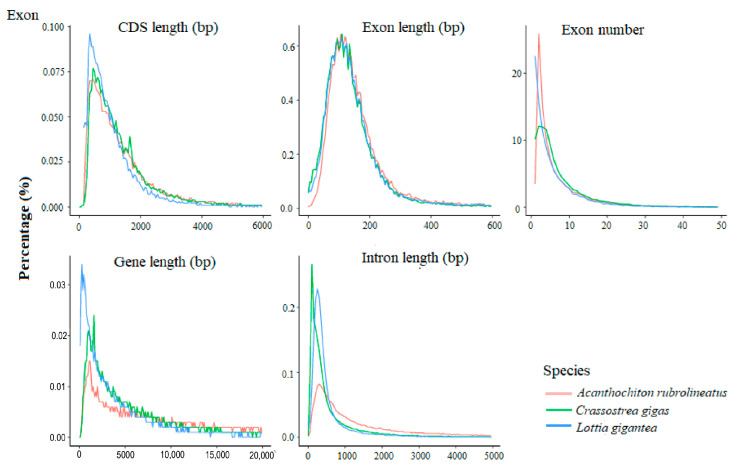
Comparison of gene structure among *A. rubrolineatus* and two related species of *C. gigas* and *L. gigantea* according to their public database source.

The genome was evaluated by BUSCO, and based on the metazoa_odb9 database, the integrity of the genome assembly, especially for those regions containing conserved genes, was predicted. Genome integrity reached 95.7%, indicating good assembly quality. The number of genes in *A. rubrolineatus* was higher than that in *Crassostrea gigas* and *Lottia gigantea* (Figure 4). To investigate genome characteristics, such as GC (guanine-cytosine) content, we analyzed the GC distribution in the genome with a sliding-window method. The peak value for the GC content was 40.10% (Table 2). We used tools such as Samtools to remove repeated reads and sort the chromosome coordinates of the Burrows-Wheeler Aligner (BWA) alignment results. We then carried out single-nucleotide polymorphism (SNP) calling and filtered the original results. We ultimately obtained a SNP value of 0.51%, of which the heterozygosis SNP ratio was 0.50% and the homozygosis SNP ratio was 0.01%. This overall SNP value was lower than the 1.30% found in *C. gigas,* but was about four times higher than that found in humans (0.14%) [51]. At the same time, the low proportion of homozygosis SNPs also indicated that the genome assembly error rate was low. In summary, these results indicated that we acquired a high-quality *A. rubrolineatus* genome.

### 3.3. Genome Annotation

Repetitive sequences within the *A. rubrolineatus* genome were identified through four different methods, which resulted in identification of ~636.61 Mb of repeated sequences that accounted for 58.88% of the assembled genome (Table 4). Among the repeated sequences, 52.92% (~572.10 Mb) were TEs. The TEs could be further divided into four main types, including short interspersed nuclear elements (SINEs) for 0.67% of the genome (~7216.09 kb), long interspersed nuclear elements (LINEs) for 14.22% of the genome (~153.78 Mb), DNA elements for 25.65% of the genome (~277.31 Mb), and long terminal repeats (LTRs) for 17.53% of the genome (~189.52 Mb) (Table 5).

After masking the repeated sequences, we annotated the protein-coding genes using de novo prediction, homology-based prediction, and transcript-based prediction. We merged the results and obtained 32,291 protein-coding genes. We checked the quality of the annotated genes by comparing them with genes from several closely related species. The messenger RNA, coding sequence (CDS), exon, and intron length distributions of *A. rubrolineatus* were similar than those of the closely related species, *C. gigas* and *L. gigantea*, suggesting that the *A. rubrolineatus* annotation results were dependable (Figure 4).

**Figure 5:**
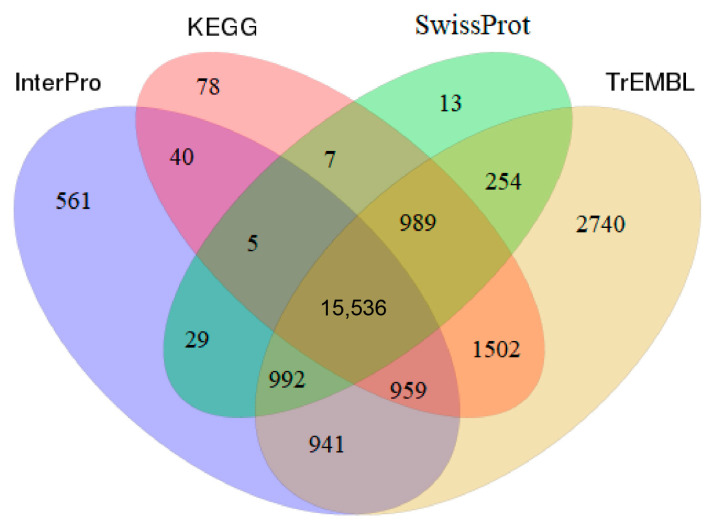
Venn diagram of annotated genes for *A. rubrolineatus* according to the respective databases.

We also performed functional annotation of the 32,291 genes with InterPro, Gene Ontology (GO), Kyoto Encyclopedia of Genes and Genomes (KEGG), SwissProt, and Translation of EMBL (TrEMBL). The highest annotation rate (74.05%) was found for TrEMBL, in which 23,912 genes were annotated (Table 6). In total, 24,645 genes (~76.32%) were annotated, indicating that most genes could be found in the public protein databases (Figure 5). Thus, we acquired sequences that represent a high-quality set of protein-coding genes for *A. rubrolineatus*.

### 3.4. Orthologous Identification and Gene Family Analysis

**Figure 6:**
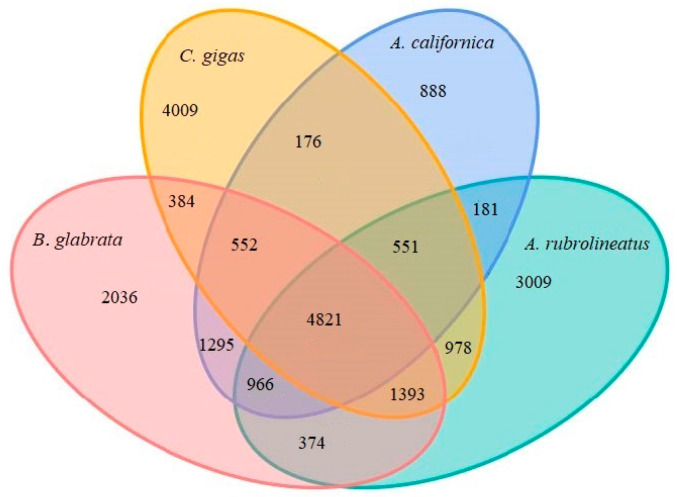
Venn diagram of the number of homologous gene families among four species, *A. rubrolineatus*, *A. californica*, *B. glabrata*, and *C. gigas*.

**Figure 7:**
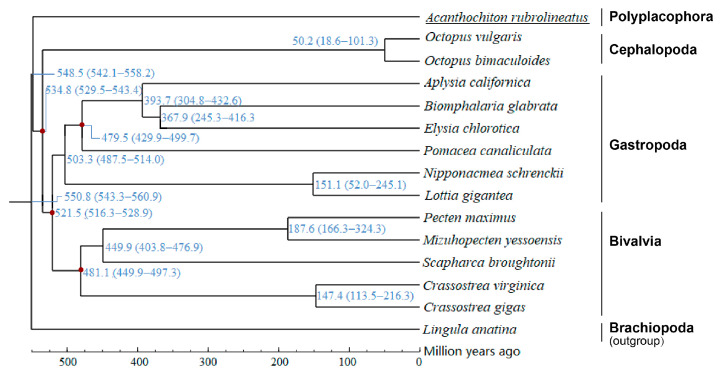
Species divergence time among the 15 species in the Mollusca. The blue number on the branch represents the estimated divergence time (in million years ago, Mya), and the 95% confidence interval for the bifurcation time is shown in parentheses. Five calibration points were selected: *O. bimaculoides* and *A. californica*, 532−582 Mya; *C. virginica* and *L. gigantea*, 520−568 Mya; *P. maximus* and *C. gigas*, 395−551 Mya; *O. vulgaris* and *B. glabrata*, 532−582 Mya; and *E. chlorotica* and *P. canaliculata*, 334−489 Mya. (Indicated by red dots in the figure, among them, the calibration points of *O. bimaculoides* and *A. californica* overlap with those of *O. vulgaris* and *B. glabrata*, and four markers are visible in the figure, Blue color indicates the interval of differentiation time).

For comparative genomics analysis of *A. rubrolineatus*, we analyzed the orthologous gene relationships among several species, including *Aplysia californica*, *B. glabrata*, *C. gigas*, *Crassostrea virginica*, *Elysia chlorotica*, *L. gigantea*, *Mizuhopecten yessoensis*, *Nipponacmea schrenckii*, *Octopus bimaculoides*, *Octopus vulgaris*, *Pecten maximus*, *Pomacea canaliculata*, *Scapharca broughtonii*, and *Lingula anatine* using OrthoMCL. A total of 34597 gene families were obtained, including 531 single copy gene families shared by all species (Table 7). We then performed gene family analysis among four related species, *A. rubrolineatus*, *A. californica*, *B. glabrata*, and *C. gigas*, and found 4821 gene families were shared among them, with 3009 gene families unique to *A. rubrolineatus* (Figure 6). Contrary to expectations, the number of gene families in common between *A. rubrolineatus* and *C. gigas* and *B. glabrata* (7743 and 7554, respectively) was higher than that between *C. gigas* and *B. glabrata* (7150) (Figure 6). This suggests that they are closer to each other in the phylogenetic tree, but a large number of gene translocations and rearrangements have occurred between them, which is also confirmed by the presence of a large number of transposon sequences in the genome of *A. rubrolineatus* (Figure 7 and Figure S1).

### 3.5. Phylogenetic Relationships and Divergence Time

Through the cluster analysis of gene families, 531 single-copy gene families were obtained in all 15 species, and these single-copy gene families were used to reconstruct phylogenetic trees. Phylogenetic analysis of the 531 highly conserved single-copy genes suggests that *A. rubrolineatus* evolved somewhere between Brachiopod and other mollusks, but they diverged earlier than other mollusks (Figure 7). The phylogenetic positions of the remaining species are similar to those reported. Five calibration points were selected: *O. bimaculoides* and *A. californica*, 532−582 Mya; *C. virginica* and *L. gigantea*, 520−568 Mya; *P. maximus* and *C. gigas*, 395−551 Mya; *O. vulgaris* and *B. glabrata*, 532−582 Mya; and *E. chlorotica* and *P. canaliculata*, 334−489 Mya. The correction point divergence time was obtained from the Timetree website (http://www.timetree.org/) (accessed on 2 October 2024). The divergence time for species was calculated by PAML (Phylogenetic Analysis by Maximum Likelihood) software (v 4.10.7). The results showed that the divergence time of Brachiopoda and Mollusca was ~550.8 Mya, and that of *A. rubrolineatus* and other mollusks was ~548.5 Mya (Figure 7). This finding, along with the fact that there has been no substantial change in morphology or lifestyle for several hundred million years among chitons [52], led us to hypothesize that Polyplacophora are characterized by “slow evolution”.

### 3.6. Gene Family Expansion and Contraction

**Figure 8:**
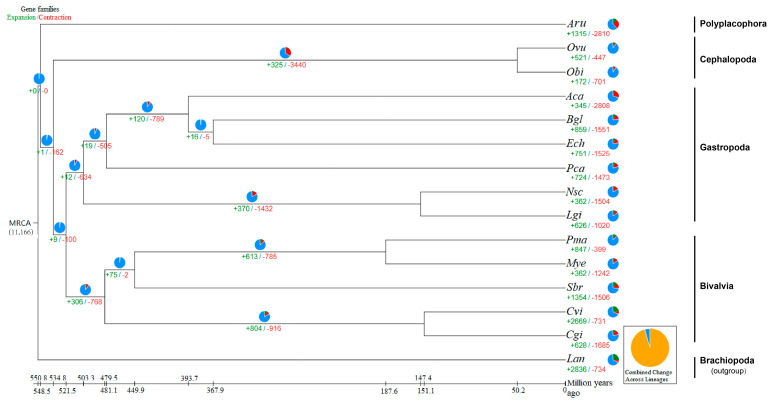
Contraction and expansion of gene families in Mollusca. *Aru*: *A. rubrolineatus*; *Aca*: *A. californica*; *Bgl*: *B. glabrata*; *Cgi*: *C. gigas*; *Cvi*: *C. virginica*; *Ech*: *E. chlorotica*; *Lgi*: *L. gigantea*; *Mye*: *M. yessoensis*; *Nsc*: *N. schrenckii*; *Obi*: *O. bimaculoides*; *Ovu*: *O. vulgaris*; *Pma*: *P. maximus*; *Pca*: *P. canaliculata*; *Sbr*: *S. broughtonii*; *Lan*: *L. anatine*. In the figure, blue represents the number of gene families of the species, green represents the number of gene families that have expanded, and red represents the number of gene families that have contracted.

**Figure 9:**
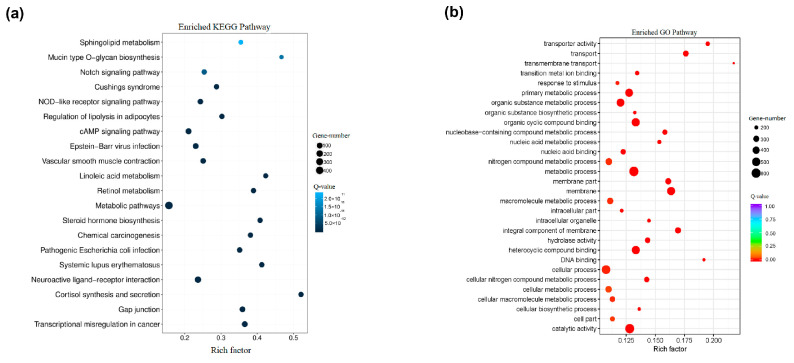
Enrichment analysis among gene families isolated from the *A. rubrolineatus* genome. (**a**) KEGG enrichment analysis for gene families. (**b**) GO enrichment analysis for gene families.

Based on the results from gene family clustering and on the phylogenetic relationship between species, gene family expansion and contraction were analyzed. After its divergence from brachiopods, *A. rubrolineatus* experienced relatively extensive gene family replacement, with a total of 1315 gene families expanding and 2810 gene families contracting (Figure 8). Among them, there were 177 gene families with significant expansion (*p* ≤ 0.05). Significantly expanded gene families were subsequently analyzed for KEGG and GO enrichment. A large number of gene families had been enriched that were related to metabolic pathways, catalytic activity, neuroactive ligand-receptor interactions, organic cyclic compound binding, heterocyclic compound binding, and so on (Figure 9). This may be because, compared with other mollusks, the teeth of chitons are replaced more frequently, which requires the continuous transportation of iron to the teeth and continuous catalytic oxidation.

With the target species of *A. rubrolineatus* as the foreground branch, *A. californica*, *B. glabrata*, *E. chlorotica*, *L. gigantea*, *N. schrenckii*, and *P. canaliculata*, were selected as the background branches. We isolated 248 genes that had undergone positive selection (*p* < 0.05), which substantially enhanced gene regulation and other functions, whereas the enzymes that played a leading role in metabolism were closely related to heterocyclic compounds (Figure S2). Here is a list of the roles of several of these genes (Table 8). These genes that encode enzymes that metabolize heterocyclic compounds may help chitons with their frequent replacement of teeth and help the less mobile Polyplacophora species adapt more effectively to changes in the environment.

## 4. Discussion

The genome of *A. rubrolineatus* expands insights into animal biology by further defining the Mollusca lineage relative to the Polyplacophora [20,53]. In this study of *A. rubrolineatus*, we found that Polyplacophora diverged very early from other mollusk classes, that the collinearity of its genome with those of other mollusks such as gastropods and bivalves was minimal, and that the *A. rubrolineatus* lineage has evolved slowly [54,55]. Our finding that genes involved in gene regulation, metabolism, and other aspects of this species were greatly expanded may be related to frequent tooth renewal and the adaptation of *A. rubrolineatus* to its environment [56,57]. Similar studies, particularly of the evolution of genomes of other Polyplacophora species, may lead to the identification of genes more closely related to mollusk ancestors, which may greatly improve our understanding of the evolution of mollusks. By constructing the genome of *A. rubrolineatus* at the chromosome level, this study created eight chromosome-level scaffolds. This finding aligns perfectly with earlier karyotype descriptions of the species. According to Odierna et al. (2008) [58], the diploid karyotype for *A. rubrolineatus* was 2n = 16, indicating the species possesses eight chromosome pairs [58]. This karyotype description corroborates our results achieved through genome assembly. Notably, the diploid karyotype number of *A. rubrolineatus* (2n = 16) ranks among the lowest documented in the phylum Mollusca, potentially illustrating its proximity to the ancestral karyotype of mollusks. The reduced karyotype number may suggest that its chromosome structure exhibits less susceptibility to rearrangements compared to other molluscan taxa, thus rendering the chromosome complement of this species a vital reference for exploring the ancestral chromosome state of mollusks. Consequently, the chromosome-level genome assembly we achieved offers significant insights for researchers in the field of malacology [59].

The unique mineralization mechanism of the radula of Polyplacophora species was a valuable system in which to study biomineralization [60]. The genome of *A. rubrolineatus* will be an important resource for future biomineralization research [61]. Although many genes involved in mollusk shell secretion undergo rapid evolution [62], the genome data presented here should help us to identify homologous genes related to the biomineralization process of mollusks. This will allow a greater understanding of the related mineralization mechanism of the radula and its metabolic pathway in mollusks [60,63]. In addition, how the requirement for using iron is balanced against its potential oxidative damage in chitons is also a problem worth studying [64].

The issue with covariance analysis arises from insufficient covariance among several species. This situation may stem from *A. rubrolineatus* not belonging to the same phylum as these species. Over millions of years, extensive genomic rearrangements occurred, adapting to increasingly complex environments. These changes led to suboptimal covariance, which corresponds with the fact that *A. rubrolineatus* was the first to diverge, maintaining minimal morphological changes over the last million years, earning it the title of a “living fossil”. Additionally, the abundance of transposon sequences in *A. rubrolineatus* highlights this issue. Studying ancient *A. rubrolineatus* can enhance our understanding of the paleoenvironment and paleoclimate, thereby advancing our research regarding the mollusk ancestor. We also hope for the sequencing of more complete genomes from polyplacophorid species, enriching our knowledge of this group.

*A. rubrolineatus* represents the first complete genome available in a Polyplacophora species. The information it provides will improve our understanding of the innovations in this lineage and biomineralization in mollusks.

## 5. Conclusions

*A. rubrolineatus* represents the first complete chromosomal genome of a porous mollusk. Its genomic data will enhance our understanding of this species’ innovations and the process of mollusk biomineralization. By comparing the genomes of *A. rubrolineatus* with those of other mollusks, we can investigate the diversity of biomineralization mechanisms in these organisms. As the genome of *A. rubrolineatus* shows minimal covariance with those of other mollusks, such as gastropods and bivalves, this indicates significant structural differences among their genomes. These differences may reflect the varying selective pressures and adaptive changes that these species experience throughout evolution. *A. rubrolineatus* exhibits a slower evolutionary rate, likely linked to its environment and lifestyle. This makes it an optimal model for examining early differentiation and evolutionary patterns in mollusks. The genome of *A. rubrolineatus* contains a notable increase in genes associated with gene regulation, metabolism, and other functions. This increase may relate to the species’ frequent tooth renewal and its adaptability to the environment, providing insights into the adaptive evolution of mollusks. These findings will deepen our understanding of molluscan evolution and aid in constructing a more accurate molluscan evolutionary tree.

## Figures and Tables

**Table 1:** Statistics of Hi-C sequencing results for *A. rubrolineatu*.

	Read 1	Read 2
Total reads	976,986,219	976,986,219
Mapped reads	780,070,392	775,452,531
Mapping ratio	79.84%	79.37%
Valid pairs	217,297,710	
Repeat	22.24%	

**Table 2:** Genome assembly information based on PacBio sequencing results for *A. rubrolineatus*.

Seq Type	Total Number	Total Length (bp)	N50 (bp)	N90 (bp)	Max Length (bp)	Min Length (bp)	Gap Length (bp)	GC Content (%)
Contig	645	1,080,820,622	3,626,433	1,015,580	17,711,703	1192	0	40.10

**Table 3:** Genome sequence length at the chromosome level for *A. rubrolineatus*.

Chromosome	Length (bp)	Percentage
1	214,362,230	19.83%
2	191,188,979	17.68%
3	160,836,634	14.88%
4	132,791,403	12.28%
5	127,116,657	11.76%
6	127,116,657	11.53%
7	72,996,094	6.75%
8	56,866,845	5.26%
Total	1,080,790,209	99.97%
Unknown	343,913	0.03%

Total is the sum of all the sequences attached to the chromosome; unknown is the sum of the unmounted parts.

**Table 4:** Percentage of repeats identified by different programs in the *A. rubrolineatus* genome.

Identification Procedure	Repeat Size (bp)	In Genome (%)
Tandem Repeats Finder	99,550,288	9.21
Repeat Masker	43,447,818	4.02
Repeat Protein Mask	30,969,758	2.86
De novo	595,700,786	55.10
Total	636,610,905	58.88

**Table 5:** Statistics for different TEs within the *A. rubrolineatus* genome.

Type	Repbase TEs		De Novo TEs		TE Proteins		Combined TEs	
	Length (bp)	In Genome (%)	Length (bp)	In Genome (%)	Length (bp)	In Genome (%)	Length (bp)	In Genome (%)
DNA	20,575,014	1.90	269,404,062	24.92	690,732	0.063	277,310,041	25.65
LINE	13,387,329	1.24	147,748,454	13.67	16,737,027	1.55	153,783,940	14.22
SINE	653,713	0.060	6,678,876	0.62	0	0	7,216,093	0.67
LTR	10,353,482	0.96	185,733,323	17.18	13,553,849	1.25	189,524,545	17.53
Others	4834	0.00040	0	0	0	0	4834	0.00040
Unknown	0	0	11,671,308	1.08	0	0	11,671,308	1.08
Total	43,447,818	4.01	564,232,691	52.19	30,969,758	2.86	572,096,658	52.92

Repbase TEs are based on the Repbase library and use Repeat Masker software (v 4.1.5) to annotate the transposon elements of the genome; De novo TEs are predicted by RepeatModeler and LTR_FINDER, combined with the RepBase library using Uclust’s software (v 11.0.630) to integrate according to the 80–80–80 principle and then use RepeatMasker Software annotates the transposon elements of the genome; TE proteins is based on the RepBase library and uses the Repeat Protein Mask software (v 4.1.7) to annotate the transposon elements of the genome; Combined TEs is the result of integrating the above two methods and removing redundancy; Others indicates that the repeating sequence can be classified by RepeatMasker, but does not belong to the above categories; Unknown indicates that the repeating sequence cannot be classified by RepeatMasker; DNA: Deoxyribonucleic acid; LINE: long interspersed nuclear element; SINE: short interspersed nuclear element; LTR: long terminal repeat.

**Table 6:** Functional annotation of 32,291 *A. rubrolineatus* genes.

Database	Number of Annotated Genes	Percentage of Isolated Genes
SwissProt	17,824	55.20
KEGG	19,115	59.20
TrEMBL	23,912	74.05
InterProScan	19,063	59.04
GO	14,292	44.26
Total	24,645	76.32

**Table 7:** Gene family clustering results among 15 species from mollusk species.

Class	Species	Number of Genes	Genes in Families	Unclustered Genes	Number of Families	Unique Families	Average Genes per Family
Polyplacophora	*A. rubrolineatus*	32,291	26,800	5491	12,273	1808	2.18
Gastropoda	*A. californica*	15,602	13,108	2494	9430	196	1.39
Gastropoda	*B. glabrata*	22,654	18,395	4259	11,821	533	1.56
Bivalvia	*C. gigas*	26,086	21,895	4191	12,864	500	1.7
Bivalvia	*C. virginica*	34,036	32,695	1341	13,336	633	2.45
Gastropoda	*E. chlorotica*	23,392	17,097	6295	12,244	402	1.4
Gastropoda	*L. gigantea*	23,324	20,826	2498	13,030	315	1.6
Bivalvia	*M. yessoensis*	22,448	20,755	1693	13,584	327	1.53
Gastropoda	*N. schrenckii*	23,878	21,035	2843	12,288	686	1.71
Cephalopoda	*O. bimaculoides*	14,855	13,786	1069	10,075	41	1.37
Cephalopoda	*O. vulgaris*	17,549	16,539	1010	10,252	113	1.61
Bivalvia	*P. maximus*	25,431	23,763	1668	13,909	248	1.71
Gastropoda	*P. canaliculata*	20,684	18,198	2486	11,003	505	1.65
Bivalvia	*S. broughtonii*	24,045	21,240	2805	12,472	576	1.7
Lingulata	*L. anatina*	25,909	22,725	3184	12,600	1434	1.8

Class

Species

Number of Genes

Genes in Families

Unclustered Genes

Number of Families

Unique Families

Average Genes per Family

**Table 8:** Functional annotation of genes that had undergone positive selection in *A. rubrolineatus*.

Gene	*p*-Value	KEGG Function
*shibie_GLEAN_10002583*	0	zinc finger protein Dzip1; K16470 zinc finger protein DZIP1
*shibie_GLEAN_10031365*	0	nucleolar GTP-binding protein 1-like; K06943 nucleolar GTP-binding protein
*shibie_GLEAN_10003691*	0	isoleucine—tRNA ligase, cytoplasmic-like; K01870 isoleucyl-tRNA synthetase[EC:6.1.1.5]
*shibie_GLEAN_10002285*	0	zinc finger HIT domain-containing protein 1; K11663 zinc finger HIT domain-containing protein 1
*shibie_GLEAN_10007267*	0	filamin-C isoform X2; K04437 filamin

## Data Availability

Data will be made available on request.

## Supplementary Materials

The following supporting information can be downloaded at: https://www.mdpi.com/article/10.3390/ani14213161/s1, Figure S1: Genome Circos plot of *A. rubrolineatus*; Figure S2: Functional enrichment analysis of positively selected genes in *A. rubrolineatus*; Table S1: Summary of Genomic Sequencing and Chromosomal-Level Assembly in Mollusca.

## References

[1] Rojas A., Urteaga D. (2011). Late Pleistocene and Holocene chitons (Mollusca, Polyplacophora) from Uruguay: Palaeobiogeography and palaeoenvironmental reconstruction in mid latitudes of the southwestern Atlantic. Geobios.

[2] Puchalski S., Eernisse D., Johnson C. (2008). The effect of sampling bias on the fossil record of chitons (Mollusca, Polyplacophora). Am. Malacol. Bull..

[3] Ni G., Kim T., Shin Y., Park J., Lee Y., Kil H.J., Park J.K. (2020). Life-history features and oceanography drive phylogeographic patterns of the chiton Acanthochitona cf. rubrolineata (Lischke, 1873) in the northwestern Pacific. PeerJ.

[4] Lowenstam H.A. (1962). Magnetite in Denticle Capping in Recent Chitons (Polyplacophora). Geol. Soc. Am. Bull..

[5] Shaw J.A., Brooker L.R., Macey D.J. (2002). Radula Tooth turnover in the chiton Acanthopleura hirtosa (Blainville, 1825) (Mollusca: Polyplacophora). Molluscan Res..

[6] Joester D., Brooker L.R. (2016). The Chiton Radula: A Model System for Versatile Use of Iron Oxides. Iron Oxides: From Nature to Applications.

[7] Kim K.-S., Macey D., Webb J., Mann S. (1989). Iron Mineralization in the Radula Teeth of the Chiton Acanthopleura hirtosa. Proc. R. Soc. London. Ser. B Biol. Sci..

[8] Shaw J.A., Macey D.J., Brooker L.R., Clode P.L. (2010). Tooth use and wear in three iron-biomineralizing mollusc species. Biol. Bull..

[9] Dixon S.J., Stockwell B.R. (2014). The role of iron and reactive oxygen species in cell death. Nat. Chem. Biol..

[10] Blakemore R. (1975). Magnetotactic bacteria. Science.

[11] Walker M.M., Diebel C.E., Haugh C.V., Pankhurst P.M., Montgomery J.C., Green C.R. (1997). Structure and function of the vertebrate magnetic sense. Nature.

[12] Roswitha Wiltschko W.W. (1995). Magnetic Orientation in Animals.

[13] Hsu C.Y., Li C.W. (1994). Magnetoreception in honeybees. Science.

[14] Fleissner G., Holtkamp-Rötzler E., Hanzlik M., Winklhofer M., Fleissner G., Petersen N., Wiltschko W. (2003). Ultrastructural analysis of a putative magnetoreceptor in the beak of homing pigeons. J. Comp. Neurol..

[15] Li Y.F., Jiang R.L., Lv H., Liu T.Y., Zhang X.Y. (2014). Preparation and characterization of petal-like superparamagnetic Fe3O4 microstructures. Rengong Jingti Xuebao/J. Synth. Cryst..

[16] Putnam N.H., Butts T., Ferrier D.E.K., Furlong R.F., Hellsten U., Kawashima T., Robinson-Rechavi M., Shoguchi E., Terry A., Yu J.-K. (2008). The amphioxus genome and the evolution of the chordate karyotype. Nature.

[17] Simakov O., Marletaz F., Cho S.-J., Edsinger-Gonzales E., Havlak P., Hellsten U., Kuo D.-H., Larsson T., Lv J., Arendt D. (2013). Insights into bilaterian evolution from three spiralian genomes. Nature.

[18] Guerra D., Bouvet K., Breton S. (2018). Mitochondrial gene order evolution in Mollusca: Inference of the ancestral state from the mtDNA of Chaetopleura apiculata (Polyplacophora, Chaetopleuridae). Mol. Phylogen. Evol..

[19] Boore J.L., Brown W.M. (1994). Complete DNA sequence of the mitochondrial genome of the black chiton, Katharina tunicata. Genetics.

[20] Irisarri I., Uribe J.E., Eernisse D.J., Zardoya R. (2020). A mitogenomic phylogeny of chitons (Mollusca: Polyplacophora). BMC Evol. Biol..

[21] Irisarri I., Eernisse D.J., Zardoya R. (2014). Molecular phylogeny of Acanthochitonina (Mollusca: Polyplacophora: Chitonida): Three new mitochondrial genomes, rearranged gene orders and systematics. J. Nat. Hist..

[22] Veale A.J., Williams L., Tsai P., Thakur V., Lavery S. (2016). The complete mitochondrial genomes of two chiton species (Sypharochiton pelliserpentis and Sypharochiton sinclairi) obtained using Illumina next generation sequencing. Mitochondrial DNA Part A.

[23] Belton J.M., McCord R.P., Gibcus J.H., Naumova N., Zhan Y., Dekker J. (2012). Hi-C: A comprehensive technique to capture the conformation of genomes. Methods.

[24] Koren S., Walenz B.P., Berlin K., Miller J.R., Bergman N.H., Phillippy A.M. (2017). Canu: Scalable and accurate long-read assembly via adaptive k-mer weighting and repeat separation. Genome Res..

[25] Walker B.J., Abeel T., Shea T., Priest M., Abouelliel A., Sakthikumar S., Cuomo C.A., Zeng Q., Wortman J., Young S.K. (2014). Pilon: An integrated tool for comprehensive microbial variant detection and genome assembly improvement. PLoS ONE.

[26] Dudchenko O., Batra S.S., Omer A.D., Nyquist S.K., Hoeger M., Durand N.C., Shamim M.S., Machol I., Lander E.S., Aiden A.P. (2017). De novo assembly of the Aedes aegypti genome using Hi-C yields chromosome-length scaffolds. Science.

[27] Price A.L., Jones N.C., Pevzner P.A. (2005). De novo identification of repeat families in large genomes. Bioinformatics.

[28] Bao W., Kojima K.K., Kohany O. (2015). Repbase Update, a database of repetitive elements in eukaryotic genomes. Mob. DNA.

[29] Stanke M., Keller O., Gunduz I., Hayes A., Waack S., Morgenstern B. (2006). AUGUSTUS: Ab initio prediction of alternative transcripts. Nucleic Acids Res..

[30] Majoros W.H., Pertea M., Salzberg S.L. (2004). TigrScan and GlimmerHMM: Two open source ab initio eukaryotic gene-finders. Bioinformatics.

[31] Burge C., Karlin S. (1997). Prediction of complete gene structures in human genomic DNA. J. Mol. Biol..

[32] Grabherr M.G., Haas B.J., Yassour M., Levin J.Z., Thompson D.A., Amit I., Adiconis X., Fan L., Raychowdhury R., Zeng Q. (2011). Full-length transcriptome assembly from RNA-Seq data without a reference genome. Nat. Biotechnol..

[33] Pertea M., Kim D., Pertea G.M., Leek J.T., Salzberg S.L. (2016). Transcript-level expression analysis of RNA-seq experiments with HISAT, StringTie and Ballgown. Nat. Protoc..

[34] Campbell M.A., Haas B.J., Hamilton J.P., Mount S.M., Buell C.R. (2006). Comprehensive analysis of alternative splicing in rice and comparative analyses with Arabidopsis. BMC Genom..

[35] Doerks T., Copley R.R., Schultz J., Ponting C.P., Bork P. (2002). Systematic identification of novel protein domain families associated with nuclear functions. Genome Res..

[36] Elsik C.G., Mackey A.J., Reese J.T., Milshina N.V., Roos D.S., Weinstock G.M. (2007). Creating a honey bee consensus gene set. Genome Biol..

[37] Bairoch A., Apweiler R. (2000). The SWISS-PROT protein sequence database and its supplement TrEMBL in 2000. Nucleic Acids Res..

[38] Kanehisa M., Goto S. (2000). KEGG: Kyoto encyclopedia of genes and genomes. Nucleic Acids Res..

[39] Zhong S., Mao Y., Wang J., Liu M., Zhang M., Su Y. (2017). Transcriptome analysis of Kuruma shrimp (*Marsupenaeus japonicus*) hepatopancreas in response to white spot syndrome virus (WSSV) under experimental infection. Fish. Shellfish. Immunol..

[40] Jones P., Binns D., Chang H.Y., Fraser M., Li W., McAnulla C., McWilliam H., Maslen J., Mitchell A., Nuka G. (2014). InterProScan 5: Genome-scale protein function classification. Bioinformatics.

[41] Krzywinski M., Schein J., Birol I., Connors J., Gascoyne R., Horsman D., Jones S.J., Marra M.A. (2009). Circos: An information aesthetic for comparative genomics. Genome Res..

[42] Edgar R.C. (2004). MUSCLE: Multiple sequence alignment with high accuracy and high throughput. Nucleic Acids Res..

[43] Stamatakis A. (2014). RAxML version 8: A tool for phylogenetic analysis and post-analysis of large phylogenies. Bioinformatics.

[44] Jones D.T., Taylor W.R., Thornton J.M. (1992). The rapid generation of mutation data matrices from protein sequences. Comput. Appl. Biosci..

[45] Yang Z. (1997). PAML: A program package for phylogenetic analysis by maximum likelihood. Comput. Appl. Biosci..

[46] Sole-Cava A.M., Thorpe J.P. (1991). High levels of genetic variation in natural populations of marine lower invertebrates. Biol. J. Linn. Soc..

[47] Zhang G., Fang X., Guo X., Li L., Luo R., Xu F., Yang P., Zhang L., Wang X., Qi H. (2012). The oyster genome reveals stress adaptation and complexity of shell formation. Nature.

[48] Wang S., Zhang J., Jiao W., Li J., Xun X., Sun Y., Guo X., Huan P., Dong B., Zhang L. (2017). Scallop genome provides insights into evolution of bilaterian karyotype and development. Nat. Ecol. Evol..

[49] Powell D., Subramanian S., Suwansa-ard S., Zhao M., O’Connor W., Raftos D., Elizur A. (2018). The genome of the oyster Saccostrea offers insight into the environmental resilience of bivalves. DNA Res..

[50] Thai B.T., Lee Y.P., Gan H.M., Austin C.M., Croft L.J., Trieu T.A., Tan M.H. (2019). Whole Genome Assembly of the Snout Otter Clam, Lutraria rhynchaena, Using Nanopore and Illumina Data, Benchmarked Against Bivalve Genome Assemblies. Front. Genet..

[51] Guo X., He Y., Zhang L., Lelong C., Jouaux A. (2015). Immune and stress responses in oysters with insights on adaptation. Fish. Shellfish. Immunol..

[52] Jackson D.J., McDougall C., Green K., Simpson F., Wörheide G., Degnan B.M. (2006). A rapidly evolving secretome builds and patterns a sea shell. BMC Biol..

[53] Varney R.M., Yap-Chiongco M.K., Mikkelsen N.T., Kocot K.M. (2022). Genome of the lepidopleurid chiton Hanleya hanleyi (Mollusca, Polyplacophora). F1000Res.

[54] Feng J., Miao J., Ye Y., Li J., Xu K., Guo B., Yan X. (2022). Insights into the Deep Phylogeny and Novel Divergence Time Estimation of Patellogastropoda from Complete Mitogenomes. Genes.

[55] Lee Y., Kwak H., Shin J., Kim S.C., Kim T., Park J.K. (2019). A mitochondrial genome phylogeny of Mytilidae (Bivalvia: Mytilida). Mol. Phylogenet Evol..

[56] Lockwood B.L., Connor K.M., Gracey A.Y. (2015). The environmentally tuned transcriptomes of Mytilus mussels. J. Exp. Biol..

[57] Lian S., Zhao L., Xun X., Lou J., Li M., Li X., Wang S., Zhang L., Hu X., Bao Z. (2019). Genome-Wide Identification and Characterization of SODs in Zhikong Scallop Reveals Gene Expansion and Regulation Divergence after Toxic Dinoflagellate Exposure. Mar. Drugs.

[58] Odierna G., Aprea G., Barucca M., Biscotti M.A., Canapa A., Capriglione T., Olmo E. (2008). Karyology of the Antarctic chiton Nuttallochiton mirandus (Thiele, 1906) (Mollusca: Polyplacophora) with some considerations on chromosome evolution in chitons. Chromosome Res..

[59] Rausch P., Rühlemann M., Hermes B.M., Doms S., Dagan T., Dierking K., Domin H., Fraune S., von Frieling J., Hentschel U. (2019). Comparative analysis of amplicon and metagenomic sequencing methods reveals key features in the evolution of animal metaorganisms. Microbiome.

[60] Varney R.M., Speiser D.I., McDougall C., Degnan B.M., Kocot K.M. (2021). The Iron-Responsive Genome of the Chiton Acanthopleura granulata. Genome Biol. Evol..

[61] Marin F., Le Roy N., Marie B. (2012). The formation and mineralization of mollusk shell. Front. Biosci. (Schol. Ed.).

[62] Ramos-Silva P., Wall-Palmer D., Marlétaz F., Marin F., Peijnenburg K. (2021). Evolution and biomineralization of pteropod shells. J. Struct. Biol..

[63] Lu H.K., Huang C.M., Li C.W. (1995). Translocation of ferritin and biomineralization of goethite in the radula of the limpet Cellana toreuma reeve. Exp. Cell Res..

[64] Moroz L.L., Gillette R. (1995). From Polyplacophora to Cephalopoda: Comparative analysis of nitric oxide signalling in mollusca. Acta Biol. Hung..

