# Supplementary material for: A Chromosome-Level Genome Assembly of Chiton Acanthochiton rubrolineatus (Chitonida, Polyplacophora, Mollusca)
Source: Animals (Basel). 2024 Nov 4;14(21):3161. doi: 10.3390/ani14213161 (PMC11545220; doi:10.3390/ani14213161)
Supplement: Supplementary file 1 [file animals-14-03161-s001.zip › animals-3162276-supplementary.pdf]

animals

MDPI

Supplementary Materials

A chromosome-level genome assembly of chiton *Acanthochiton rubrolineatus* (Chitonida, Polyplacophora, Mollusca)

Jiangyong Qu [1,†], Xiaofei Lu [1,†], Chenen Tu [1], Fuyang He [1], Sutao Li [1], Dongyue Gu [1], Shuang Wang [1], Zhikai Xing [1], Li Zheng [2], Xumin Wang [1,*], Lijun Wang [1,*]

1 College of Life Science, Yantai University, Yantai, Shandong, 264005, China; qjy@ytu.edu.cn (J.Q.); luxiaofei18@outlook.com (X.L.); 18055456097@163.com (C.T.); 19554539611@163.com (F.H.); z123xcvbnm0709@163.com (L.S.); 19554539562@163.com (G.D.); wangshuang0456@126.com (S.W.); xingzhk@ytu.edu.cn (Z.X.); wangxm@ytu.edu.cn (X.W.); wanglijun@ytu.edu.cn (L.J.)

2 First Institute of Oceanography, Ministry of Natural Resources, Qingdao, Shandong, 266061, China. zhengli@fio.org.cn (Z.L.)

† The two authors contributed equally to this paper.

* Correspondence: wangxm@ytu.edu.cn (X.W.); wanglijun@ytu.edu.cn (L.J.)

This file contains supplementary 1 table and 1 Figure:

Table S1. Summary of Genomic Sequencing and Chromosomal-Level Assembly in Mollusca

Figure. S1 Genome Circos plot of *A. rubrolineatus*.

Figure S2. Functional enrichment analysis of positively selected genes in *A. rubrolineatus*.

Table S1. Summary of Genomic Sequencing and Chromosomal-Level Assembly in Mollusca.

Species	chromosome/ whole-genome	lengths	NCBI Reference Sequence
Biomphalaria glabrata	chromosome1	90,404,365 bp	NC_074711.1
	chromosome2	65,242,864 bp	NC_074712.1
	chromosome7	48,536,009 bp	NC_074717.1
	chromosome3	56,616,397 bp	NC_074713.1
	chromosome5	55,047,444 bp	NC_074715.1
	chromosome10	45,784,278 bp	NC_074720.1
	chromosome4	56,610,832 bp	NC_074714.1
	chromosome8	47,236,035 bp	NC_074718.1
	chromosome17	28,844,021 bp	NC_074727.1
	chromosome11	43,100,265 bp	NC_074721.1
	chromosome12	39,291,701 bp	NC_074722.1
	chromosome9	47,122,645 bp	NC_074719.1
	chromosome6	54,851,608 bp	NC_074716.1
	chromosome18	24,813,541 bp	NC_074728.1
	chromosome16	32,813,434 bp	NC_074726.1
	chromosome15	32,883,732 bp	NC_074725.1
	chromosome14	37,700,536 bp	NC_074724.1
	chromosome13	38,961,879 bp	NC_074723.1
Crassostrea angulata	chromosome6	61,759,399 bp	NC_069116.1
	chromosome1	57,608,718 bp	NC_069111.1
	chromosome2	81,655,936 bp	NC_069112.1
	chromosome3	58,148,000 bp	NC_069113.1
	chromosome4	51,822,500 bp	NC_069114.1
	chromosome8	60,476,782 bp	NC_069118.1

	chromosome10	56,228,760 bp	NC_069120.1
	chromosome7	60,847,761 bp	NC_069117.1
	chromosome5	61,934,658 bp	NC_069115.1
	chromosome9	39,826,282 bp	NC_069119.1
Crassostrea virginica	chromosome5	98,698,416 bp	NC_035784.1
	chromosome4	59,691,872 bp	NC_035783.1
	chromosome1	65,668,440 bp	NC_035780.1
	chromosome3	77,061,148 bp	NC_035782.1
	chromosome2	61,752,955 bp	NC_035781.1
	chromosome9	34,737,321 bp	NC_037593.1
	chromosome8	75,944,018 bp	NC_035787.1
	chromosome6	51,258,098 bp	NC_035785.1
	chromosome7	57,830,854 bp	NC_035786.1
	chromosome10	32,650,045 bp	NC_035789.1
Dreissena polymorpha	chromosome 1	211,287,878 bp	NC_068355.1
	chromosome2	155,660,206 bp	NC_068356.1
	chromosome3	152,547,682 bp	NC_068357.1
	chromosome4	145,370,454 bp	NC_068358.1
	chromosome9	106,873,626 bp	NC_068363.1
	chromosome10	90,528,778 bp	NC_068364.1
	chromosome16	51,057,412 bp	NC_068370.1
	chromosome5	127,705,040 bp	NC_068359.1
	chromosome11	87,507,468 bp	NC_068365.1
	chromosome8	107,691,933 bp	NC_068362.1
	chromosome12	77,012,831 bp	NC_068366.1
	chromosome7	111,411,237 bp	NC_068361.1
	chromosome15	66,979,997 bp	NC_068369.1
	chromosome14	75,447,393 bp	NC_068368.1
	chromosome6	117,515,028 bp	NC_068360.1
	chromosome13	75,790,364 bp	NC_068367.1
Gigantopelta aegis	chromosome9	84,917,595 bp	NC_054707.1
	chromosome4	120,622,278 bp	NC_054702.1
	chromosome6	102,884,749 bp	NC_054704.1
	chromosome10	91,665,651 bp	NC_054708.1
	chromosome3	96,789,575 bp	NC_054701.1
	chromosome2	54,566,076 bp	NC_054700.1
	chromosome8	81,591,406 bp	NC_054706.1
	chromosome14	59,883,994 bp	NC_054712.1
	chromosome5	40,821,028 bp	NC_054703.1
	chromosome7	47,477,099 bp	NC_054705.1
	chromosome1	47,317,128 bp	NC_054699.1
	chromosome11	35,397,884 bp	NC_054709.1
	chromosome12	53,390,639 bp	NC_054710.1
	chromosome15	46,388,186 bp	NC_054713.1
	chromosome13	42,253,053 bp	NC_054711.1
Haliotis asinina	chromosome3	89,013,229 bp	NC_090282.1
	chromosome5	79,603,559 bp	NC_090284.1
	chromosome9	66,300,187 bp	NC_090288.1
	chromosome12	58,286,999 bp	NC_090291.1
	chromosome2	101,978,182 bp	NC_090281.1
	chromosome8	66,935,639 bp	NC_090287.1

	chromosome14	55,702,281 bp	NC_090293.1
	chromosome7	67,096,918 bp	NC_090286.1
	chromosome1	105,963,769 bp	NC_090280.1
	chromosome13	55,744,664 bp	NC_090292.1
	chromosome6	71,828,453 bp	NC_090285.1
	chromosome15	52,850,021 bp	NC_090294.1
	chromosome4	83,060,790 bp	NC_090283.1
	chromosome10	59,771,656 bp	NC_090289.1
	chromosome16	49,714,875 bp	NC_090295.1
	chromosome11	58,626,679 bp	NC_090290.1
	chromosome 6	82,493,220 bp	NC_088300.1
	chromosome1	96,433,062 bp	NC_088295.1
	chromosome7	60,359,117 bp	NC_088301.1
	chromosome8	58,388,612 bp	NC_088302.1
	chromosome10	36,638,032 bp	NC_088304.1
	chromosome9	37,343,639 bp	NC_088303.1
Liolophura japonica	chromosome2	28,395,274 bp	NC_088296.1
	chromosome13	30,102,928 bp	NC_088307.1
	chromosome5	19,479,325 bp	NC_088299.1
	chromosome3	27,489,247 bp	NC_088297.1
	chromosome12	31,077,156 bp	NC_088306.1
	chromosome4	25,057,479 bp	NC_088298.1
	chromosome11	32,819,725 bp	NC_088305.1
	chromosome 5	57,274,926 bp	NC_088857.1
	chromosome 7	53,672,946 bp	NC_088859.1
	chromosome1	76,070,991 bp	NC_088853.1
	chromosome3	61,039,741 bp	NC_088855.1
Magallana gigas	chromosome4	57,946,171 bp	NC_088856.1
	chromosome6	56,905,015 bp	NC_088858.1
	chromosome2	61,469,542 bp	NC_088854.1
	chromosome8	51,133,819 bp	NC_088860.1
	chromosome9	50,364,239 bp	NC_088861.1
	chromosome10	37,310,742 bp	NC_088862.1
	chromosome3	104,084,471 bp	NC_069363.1
	chromosome13	81,946,364 bp	NC_069373.1
	chromosome17	73,516,942 bp	NC_069377.1
	chromosome6	91,270,759 bp	NC_069366.1
	chromosome4	100,968,995 bp	NC_069364.1
	chromosome9	85,987,162 bp	NC_069369.1
	chromosome16	74,149,818 bp	NC_069376.1
	chromosome2	120,499,105 bp	NC_069362.1
Mercenaria mercenaria	chromosome8	86,514,381 bp	NC_069368.1
	chromosome10	82,914,371 bp	NC_069370.1
	chromosome1	121,110,697 bp	NC_069361.1
	chromosome12	82,356,740 bp	NC_069372.1
	chromosome5	97,832,188 bp	NC_069365.1
	chromosome18	64,330,741 bp	NC_069378.1
	chromosome19	46,480,821 bp	NC_069379.1
	chromosome15	74,526,284 bp	NC_069375.1
	chromosome11	82,432,825 bp	NC_069371.1
	chromosome14	76,376,716 bp	NC_069374.1

	chromosome7	88,078,429 bp	NC_069367.1
	chromosome4	82,781,202 bp	NC_069125.1
	chromosome2	71,556,252 bp	NC_069123.1
	chromosome3	93,038,574 bp	NC_069124.1
	chromosome8	75,813,843 bp	NC_069129.1
	chromosome10	74,570,910 bp	NC_069131.1
	chromosome12	70,040,107 bp	NC_069133.1
	chromosome13	69,354,228 bp	NC_069134.1
	chromosome5	80,778,335 bp	NC_069126.1
Mya arenaria	chromosome14	64,373,224 bp	NC_069135.1
	chromosome11	71,350,902 bp	NC_069132.1
	chromosome16	52,865,736 bp	NC_069137.1
	chromosome17	56,306,838 bp	NC_069138.1
	chromosome6	83,634,289 bp	NC_069127.1
	chromosome7	71,406,691 bp	NC_069128.1
	chromosome15	58,022,990 bp	NC_069136.1
	chromosome9	76,804,856 bp	NC_069130.1
	chromosome1	63,369,314 bp	NC_069122.1
	chromosome 4	96,324,366 bp	NC_086376.1
	chromosome1	113,486,924 bp	NC_086373.1
	chromosome6	91,445,414 bp	NC_086378.1
	chromosome2	103,707,636 bp	NC_086374.1
	chromosome7	85,406,108 bp	NC_086379.1
	chromosome3	94,312,406 bp	NC_086375.1
Mytilus trossulus	chromosome9	82,295,107 bp	NC_086381.1
	chromosome11	71,253,669 bp	NC_086383.1
	chromosome5	88,129,055 bp	NC_086377.1
	chromosome14	81,773,519 bp	NC_086386.1
	chromosome10	78,455,819 bp	NC_086382.1
	chromosome13	63,222,996 bp	NC_086385.1
	chromosome8	80,675,551 bp	NC_086380.1
	chromosome12	60,198,110 bp	NC_086384.1
	chromosome1	199,874,329 bp	NC_068981.1
	chromosome6	117,080,279 bp	NC_068986.1
	chromosome15	58,155,575 bp	NC_068995.1
	chromosome2	192,492,730 bp	NC_068982.1
	chromosome5	127,476,059 bp	NC_068985.1
	chromosome25	35,489,836 bp	NC_069005.1
	chromosome7	110,520,659 bp	NC_068987.1
	chromosome9	96,881,196 bp	NC_068989.1
	whole-genome	147,805,816 bp	NC_068984.1
Octopus bimaculoides	chromosome11	80,868,371 bp	NC_068991.1
	chromosome21	40,062,750 bp	NC_069001.1
	chromosome22	37,717,999 bp	NC_069002.1
	chromosome4	168,056,177 bp	NC_068983.1
	chromosome14	60,576,134 bp	NC_068994.1
	chromosome8	97,793,173 bp	NC_068988.1
	chromosome16	57,524,431 bp	NC_068996.1
	chromosome18	55,557,504 bp	NC_068998.1
	chromosome24	36,166,163 bp	NC_069004.1
	chromosome10	94,598,374 bp	NC_068990.1

	chromosome13	65,066,837 bp	NC_068993.1
	chromosome28	18,637,145 bp	NC_069008.1
	chromosome19	55,137,729 bp	NC_068999.1
	chromosome27	22,553,649 bp	NC_069007.1
	chromosome20	47,890,971 bp	NC_069000.1
	chromosome26	26,644,807 bp	NC_069006.1
	chromosome17	57,421,550 bp	NC_068997.1
	chromosome30	10,083,081 bp	NC_069010.1
	chromosome29	11,991,813 bp	NC_069009.1
Ostrea edulis	chromosome 14	94,441,566 bp	NC_079167.1
	chromosome 19	75,578,129 bp	NC_079172.1
	chromosome1	112,480,954 bp	NC_079164.1
	chromosome2	109,476,225 bp	NC_079165.1
	chromosome5	94,306,699 bp	NC_079168.1
	chromosome6	93,534,300 bp	NC_079169.1
	chromosome7	88,744,346 bp	NC_079170.1
	chromosome8	77,126,126 bp	NC_079171.1
	chromosome3	98,268,724 bp	NC_079166.1
	chromosome10	48,989,042 bp	NC_079173.1
Patella vulgata	chromosome2	94,032,750 bp	NC_065880.2
	chromosome3	90,894,639 bp	NC_065881.2
	chromosome4	87,393,929 bp	NC_065882.2
	chromosome6	75,114,587 bp	NC_065884.2
	chromosome5	80,768,876 bp	NC_065883.2
	chromosome1	95,737,551 bp	NC_065879.2
	chromosome7	68,900,667 bp	NC_065885.2
	chromosome8	54,963,775 bp	NC_065886.2
	chromosome9	47,190,821 bp	NC_065887.2
Pecten maximus	chromosome9	45,552,881 bp	NC_047023.1
	chromosome12	42,360,788 bp	NC_047026.1
	chromosome1	60,076,705 bp	NC_047015.1
	chromosome11	44,189,035 bp	NC_047025.1
	chromosome2	55,138,983 bp	NC_047016.1
	chromosome10	44,824,366 bp	NC_047024.1
	chromosome6	47,357,467 bp	NC_047020.1
	chromosome8	46,176,750 bp	NC_047022.1
	chromosome7	46,316,487 bp	NC_047021.1
	chromosome5	51,398,101 bp	NC_047019.1
	chromosome17	33,917,975 bp	NC_047031.1
	chromosome3	53,454,838 bp	NC_047017.1
	chromosome4	52,693,527 bp	NC_047018.1
	chromosome18	33,254,064 bp	NC_047032.1
	chromosome19	32,483,354 bp	NC_047033.1
	chromosome16	37,770,382 bp	NC_047030.1
	chromosome14	38,909,756 bp	NC_047028.1
	chromosome15	38,817,130 bp	NC_047029.1
	chromosome13	39,606,779 bp	NC_047027.1
Pomacea canaliculata	whole-genome	23,556,918 bp	NC_037602.1
	whole-genome	45,157,847 bp	NC_037591.1
	whole-genome	34,737,321 bp	NC_037593.1
	whole-genome	34,494,602 bp	NC_037594.1

	whole-genome	43,189,359 bp	NC_037592.1
	whole-genome	45,418,568 bp	NC_037590.1
	whole-genome	31,531,291 bp	NC_037595.1
	whole-genome	27,161,898 bp	NC_037598.1
	whole-genome	26,524,634 bp	NC_037599.1
	whole-genome	23,662,357 bp	NC_037601.1
	whole-genome	24,344,826 bp	NC_037600.1
	whole-genome	29,527,706 bp	NC_037597.1
	whole-genome	19,366,143 bp	NC_037603.1
	whole-genome	30,920,076 bp	NC_037596.1
Octopus sinens	whole-genome	77,502,973 bp	NC_043007.1
	whole-genome	120,472,873 bp	NC_043002.1
	whole-genome	213,406,131 bp	NC_042997.1
	whole-genome	207,928,902 bp	NC_042998.1
	whole-genome	25,544,736 bp	NC_043022.1
	whole-genome	28,926,792 bp	NC_043021.1
	whole-genome	135,913,566 bp	NC_043001.1
	whole-genome	117,540,060 bp	NC_043003.1
	whole-genome	105,892,736 bp	NC_043006.1
	whole-genome	59,466,961 bp	NC_043012.1
	whole-genome	107,269,306 bp	NC_043004.1
	whole-genome	164,836,235 bp	NC_043000.1
	whole-genome	174,423,281 bp	NC_042999.1
	whole-genome	30,097,204 bp	NC_043018.1
Octopus sinensis	whole-genome	21,331,139 bp	NC_043023.1
	whole-genome	72,296,471 bp	NC_043009.1
	whole-genome	34,119,079 bp	NC_043017.1
	whole-genome	59,356,462 bp	NC_043013.1
	whole-genome	68,403,164 bp	NC_043010.1
	whole-genome	67,901,367 bp	NC_043011.1
	whole-genome	109,703,381 bp	NC_043005.1
	whole-genome	32,879,656 bp	NC_043019.1
	whole-genome	20,263,137 bp	NC_043024.1
	whole-genome	32,976,114 bp	NC_043016.1
	whole-genome	55,351,117 bp	NC_043014.1
	whole-genome	31,203,755 bp	NC_043020.1
	whole-genome	17,062,404 bp	NC_043025.1
	whole-genome	15,106,159 bp	NC_043026.1

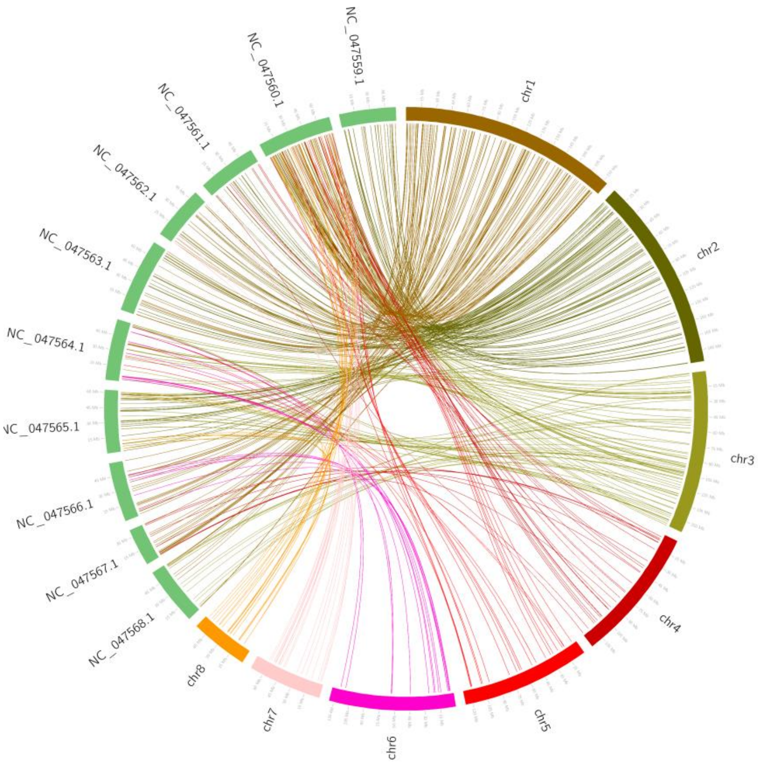

Figure S1. Genome-wide covariance analysis of *C. gigas.*

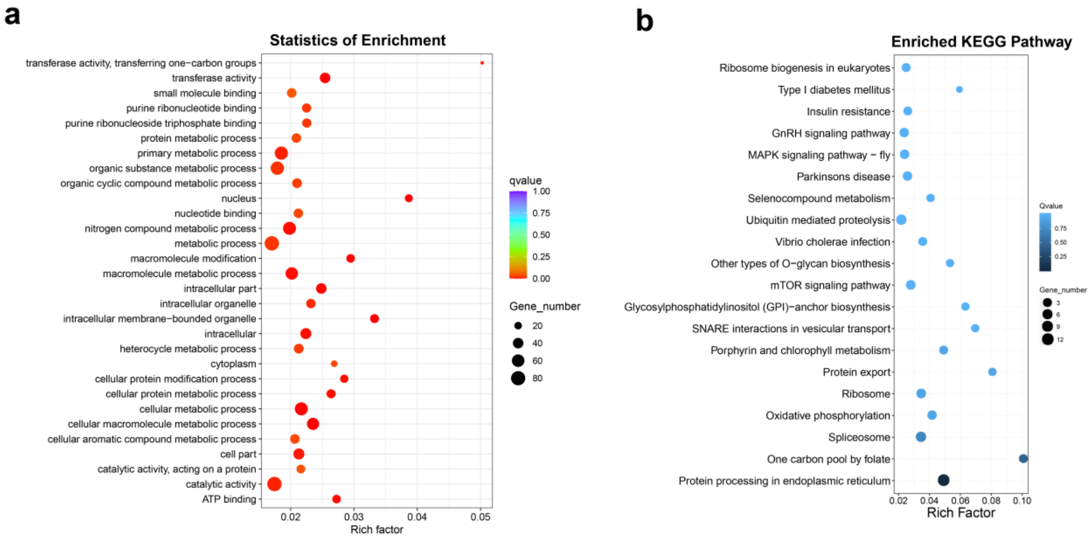

Figure S2. Functional enrichment analysis of positively selected genes in *A. rubrolineatus*. (a) GO enrichment analysis for gene families. (b) KEGG enrichment analysis for gene families.
